# Effect of methyl methanesulfonate on hsp70 expression and tissue damage in the third instar larvae of transgenic *Drosophila melanogaster (hsp70-lacZ) Bg* ^*9*^

**DOI:** 10.2478/v10102-011-0025-7

**Published:** 2011-09

**Authors:** Vineet Kumar, Gulshan Ara, Mohammad Afzal, Yasir Hasan Siddique

**Affiliations:** Drosophila Transgenics Laboratory, Section of Genetics, Department of Zoology, Aligarh Muslim University, Aligarh-202002, UP, Aligarh, INDIA

**Keywords:** Drosophila melanogaster (hsp70-lacZ) Bg9, Methyl methanesulfonate, hsp70

## Abstract

Methyl methanesulfonate (MMS) is an anti-carcinogenic drug and its toxicity has been reported in various experimental models. The hsp70s are a family of ubiquitously expressed heat shock proteins. In the recent years, hsp70 has been considered to be one of the candidate genes for predicting cytotoxicity against environmental chemicals. Nowadays emphasis is given to the use of alternatives to mammals in testing, research and education. The European Centre for the Validation of Alternative Methods (EVCAM) has recommended the use of *Drosophila* as an alternative model for scientific studies. Almost all living organisms possess proteins with a similar structure to that of hsp70s. In the present study, the toxicity of MMS was evaluated by quantifying hsp70 expression and tissue damage in the third instar larvae of transgenic *Drosophila melanogaster (hsp70-lacZ) Bg* ^*9*^, at different doses and hours of exposure. We studied the effect of 0.25, 0.50, 0.75 and 1.0 µl/ml of MMS at 2, 4, 24 and 48 hours of exposure on hsp70 expression by using the soluble O-nitrophenyl-β-D-galactopyranoside (ONPG) assay and on establishing the tissue damage by the Trypan blue exclusion assay in the third instar larvae of transgenic *Drosophila melanogaster (hsp70-lacZ) Bg* ^*9*^. A dose-dependent increase in the expression of hsp70 was observed at 0.25, 0.50, and 0.75 µl/ml of MMS compared to the control. At the highest dose, *i.e.* 1.0 µl/ml of MMS, the activity of hsp70 was decreased due to tissue damage.

## Introduction

The hsp70s are a family of ubiquitously expressed heat shock proteins. Almost all living organisms possess proteins with structure similar to that of hsp70s. The hsp70s are an important part of the cell machinery for protein folding and they help to protect cells from stress (Tavaria *et al.,* 1996; Morano, 2007). Members of the hsp70 family are strongly up-regulated by heat stress and toxic chemicals, particularly heavy metals such as arsenic, cadmium, copper, mercury, etc. Hsp70 was originally discovered by F.M. Ritossa in the 1960s when a lab worker accidentally boosted the incubation temperature of *Drosophila* (fruit flies). While examining the chromosomes, Ritossa found a “puffing pattern” that indicated elevated gene transcription of an unknown protein (Ritossa, 1996). This was later described as the “Heat Shock Response” and the proteins were termed as “Heat Shock Proteins” (hsps). The argument to use measurements of this stress response as a biomarker is based on the mode of induction of the *hsp70* gene(s) and the corresponding protein class, hsp70, which so far represents the best investigated family of stress proteins. The role of hsp70 in intracellular protein folding and the transmembrane protein passage is based on their capability to bind uncoiled polypeptide chain (Kohler *et al.,* 1998). Genes encoding heat shock proteins are highly conserved and many of their products can be assigned to families on the basis of sequence homology and molecular weight. In an un-stressed cell, hsp acts in successful folding, assembly, intracellular localization, secretion, regulation and degradation of other proteins (Fonager *et al.,* 2002). Under conditions in which protein folding is perturbed or proteins begin to unfold and denature, hsp assists in protein refolding, in protecting cellular systems against protein damage, in solubilizing aggregates to some extent, in sequestering overloaded and damaged protein to degradation machinery (Fonager *et al.,* 2002). Under stressful conditions, all living organism respond by synthesizing heat shock proteins (HSPs) (Nover, 1984; 1991). HSPs function as molecular chaperons that prevent cellular damage (Bennett & Waters, 2000). In the recent years, *hsp70* has been considered to be one of the candidate genes for predicting cytotoxicity against environmental chemicals (Bierkens, 2000; Mukhopadhyay *et al.,* 2003; Mukhopadhyay *et al.,* 2002; Lis *et al.,* 1983; Siddique *et al.,* 20112011a, b).

Methyl methanesulfonate (MMS) is a quiet stable molecule, but the presence of oxidizing agents, acids, alkali and excess heat may lead to its instability. Exposure to MMS appears to be limited to laboratory research personnel (HSDB, 2000). It is classified not only as a carcinogen but also as a mutagenic agent for bacteria and yeast (alkylating agent). It has also been reported to cause developmental toxicity (HSDB, 2000). The American Conference of Governmental Industrial Hygienists (ACGIH 1997) has not proposed any occupational exposure limit for MMS in workplace air and no international guidelines for MMS in drinking-water have been established (WHO, 1993). MMS methylates DNA on N^7^-deoxyguanine and N^3^-deoxyadenine. Originally, this action was believed to directly cause double-stranded DNA breaks, because homologous recombination-deficient cells are particularly vulnerable to the effects of MMS (Lundin *et al.,* 2005). MMS is used experimentally as a mutagen, teratogen, and brain carcinogen, as a research chemical, and also as a catalyst in chemical synthesis (IARC, 1974; Merck,^1989^; HSDB, 2000). Methanesulfonic acid monoesters may be used as insect and mammalian pest chemosterilants and also as a possible human male contraceptive (IARC 1974 & 1987). Most of the chemotherapeutic agents target DNA (Cozzi *et al.,* 2004). Therefore, the DNA repair status is of utmost importance for tumor sensitivity to drugs and at the same time for the protection of normal tissue. In humans the therapeutic application of MMS to cancer patients of total doses ranging from 2.8 to 800 mg/kg body weight over a period of up to 350 days led to significant gastrointestinal and hepatic toxic effects. MMS induced somatic and sex-linked mutations in *Drosophila* (Y*oda et al.,* 1982 *).* Nowadays the use of animals in toxicological research and testing has become an important issue for both science and ethics. As a result emphasis has been given to the use of alternatives to mammals in testing, research and education (Mukhopadhyay *et al.,* 2003). The European Centre for the Validation of Alternative Methods (EVCAM) has recommended the use of *Drosophila* as an alternative model for scientific studies (Festing *et al.,* 1998; Benford *et al.,* 2000). The effect of various pesticides, such as hexa chlorocyclohexane (Chowdhuri *et al.*, 1999), Chlorpyrifos (Nazir *et al.*, 2001), organophosphate compounds (Gupta *et al.*, 2007), fungicides such as captan (Nazir *et al.*, 2003), argemone oil (Mukhopadyay *et al.*, 2003), and industrial solid wastes (Siddique *et al.*, 2005), has been studied for hsp70 expression in the third instar larvae of transgenic *Drosophila melanogaster (hsp70-lacZ)Bg* ^*9*^.

**Figure 1:**
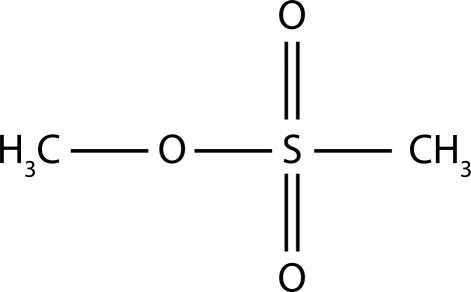
Structure of methyl methanesulfonate.

In the present study, the toxicity of different doses and hours of exposure of MMS was evaluated by quantifying the *hsp70* expression and tissue damage in the third instar larvae of transgenic *Drosophila melanogaster* *(hsp70-lacZ) Bg* ^*9*^.

## Methods

### Fly strain

A transgenic *Drosophila melanogaster* line that expresses bacterial beta-galactosidase as a response to stress was used in the present study (Lis *et al.*, 1983). In the said strain of flies, the transformation vector is inserted with a P-element, the line contains wild type *hsp70* sequence up to the *lacZ* fusion point. The flies and larvae were cultured on standard *Drosophila* food containing agar, corn meal, sugar, and yeast at 24°C±1 (Nazir *et al.,* 2003).

### Experimental design

MMS concentrations at 0.25, 0.50, 0.75 and 1.0 µl/ml of food were established. The third instar larvae were allowed to feed on them for different time intervals (2, 4, 24 and 48 hrs).

### Soluble O-nitrophenyl-β-D-galactopyranoside (ONPG) assay

The expression of *hsp70* gives the measure of cytotoxicity (Chowdhuri *et al.,* 1996Chowdhuri *et al.,* 2001). We followed the method as described by Nazir *et al.* (2003). Briefly, after washing the larvae in phosphate buffer, they were put in a microcentrifuge tube (20 larvae / tube; 5 replicates/group), permeabilized for l0 min by acetone, and incubated overnight at 37°C in 600 µl of ONPG staining buffer. Following incubation, the reaction was stopped by adding 300 µl of Na_2_CO_3_. The extent of the reaction was quantified by measuring the absorbance at 420 nm using Systronics UV/VIS Spectrophotometer 118, India.

### Trypan blue exclusion test

The extent of tissue damage in larvae caused by the exposure to different concentrations of MMS was assayed by a dye exclusion test (Krebs & Feder, 1997; Nazir *et al.,* 2003). Briefly, the internal tissues of larvae were explanted in a drop of phosphate buffer (PB), rotated in trypan blue stain for 30 min, washed thoroughly in PB, and scored immediately for dark blue staining. A total of 50 larvae per treatment (10 larvae per dose; 5 replicates per group) were scored for the trypan blue staining on an average composite index per larva: no color, 0; any blue, 1; darkly stained nuclei, 2; large patches of darkly stained cells, 3; or complete staining of most cells in the tissue, 4 (Krebs & Feder, 1997).

### Statistical analysis

Statistical analysis was carried out by Student's *t* test using commercial software statistica Soft Inc, India (2007).

## Results

**Figure 2:**
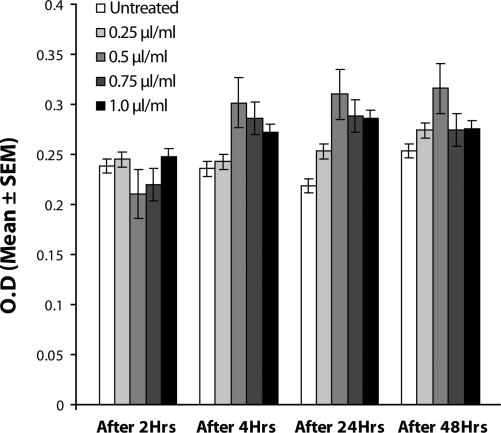
Mean absorbance value after exposure to various doses of MMS.

**Figure 3:**
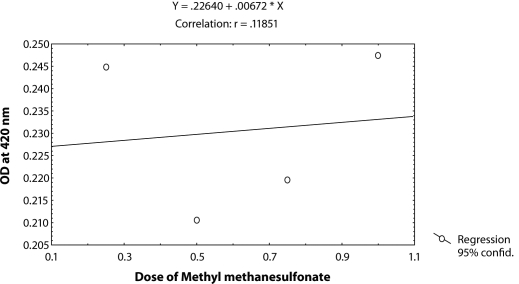
Regression analysis for β-galactosidase activity in the third instar larvae of transgenic *Drosophila melanogaster* *(hsp70-lacZ) Bg* ^*9*^ exposed to 0.25, 0.50, 0.75 and 1.0 µl/ml of MMS for 2 hrs.

**Figure 4:**
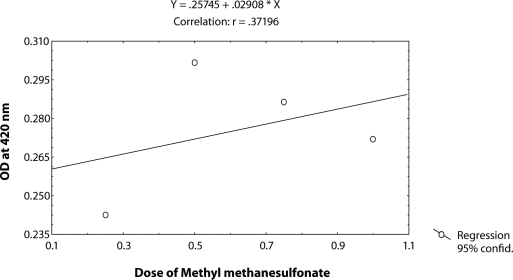
Regression analysis for β-galactosidase activity in the third instar larvae of transgenic *Drosophila melanogaster* *(hsp70-lacZ) Bg* ^*9*^ exposed to 0.25, 0.50, 0.75 and 1.0 µl/ml of MMS for 4 hrs.

**Figure 5:**
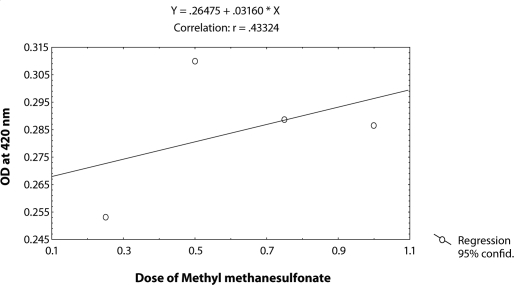
Regression analysis for β-galactosidase activity in the third instar larvae of transgenic *Drosophila melanogaster* *(hsp70-lacZ) Bg* ^*9*^ exposed to 0.25, 0.50, 0.75 and 1.0 µl/ml of MMS for 24 hrs.

**Figure 6:**
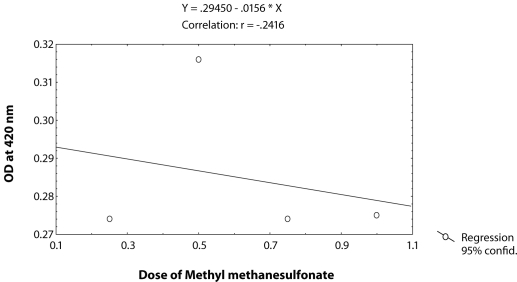
Regression analysis for β-galactosidase activity in the third instar larvae of transgenic *Drosophila melanogaster* (*hsp70-lacZ) Bg* ^*9*^ exposed to 0.25, 0.50, 0.75 and 1.0 µl/ml of MMS for 48 hrs.

**Figure 7:**
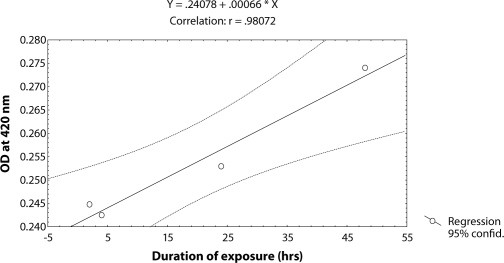
Regression analysis for β-galactosidase activity in the third instar larvae of transgenic *Drosophila melanogaster* *(hsp70-lacZ) Bg* ^*9*^ exposed to 0.25 µl/ml of MMS for 2, 4, 24 and 48 hrs.

**Figure 8:**
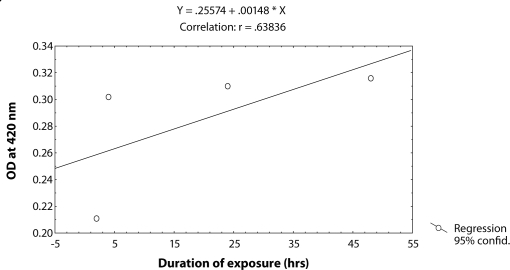
Regression analysis for β-galactosidase activity in the third instar larvae of transgenic *Drosophila melanogaster* *(hsp70-lacZ) Bg* ^*9*^ exposed to 0.50 µl/ml of MMS for 2, 4, 24 and 48 hrs.

**Figure 9:**
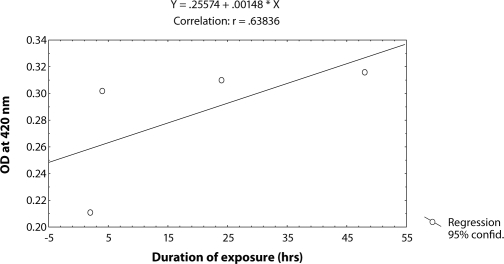
Regression analysis for β-galactosidase activity in the third instar larvae of transgenic *Drosophila melanogaster* *(hsp70-lacZ) Bg* ^*9*^ exposed to 0.75 µl/ml of MMS for 2, 4, 24 and 48 hrs.

**Figure 10:**
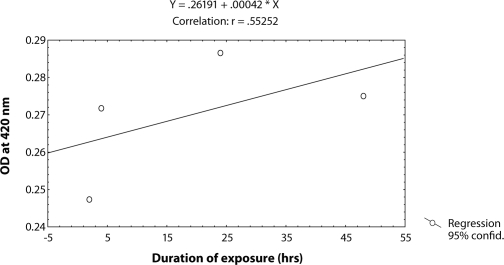
Regression analysis for β-galactosidase activity in the third instar larvae of transgenic *Drosophila melanogaster* *(hsp70-lacZ) Bg* ^*9*^ exposed to 1.0 µl/ml of MMS for 2, 4, 24 and 48 hrs.

**Figure 11:**
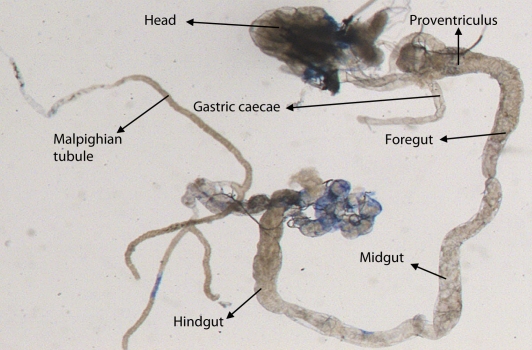
Trypan blue staining pattern in the third instar larval tissues of *D. melanogaster* *(hsp70-lacZ) Bg* ^*9*^ for 48 hrs (control).

**Figure 12:**
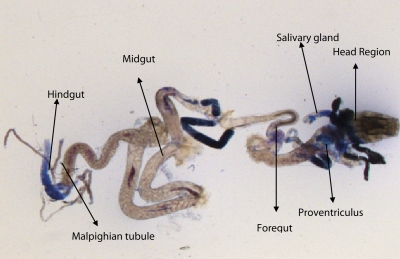
Trypan blue staining pattern in the third instar larval tissues of *D. melanogaster* *(hsp70-lacZ) Bg* ^*9*^ after exposure to 0.50 µl/ml of MMS for 48 hrs.

**Figure 13:**
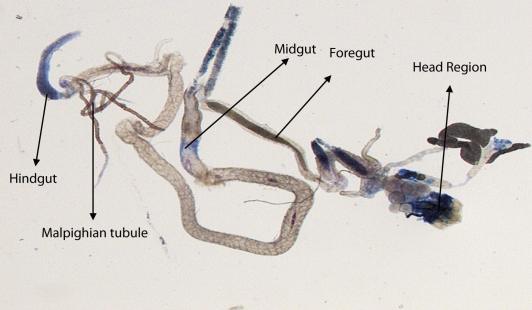
Trypan blue staining pattern in the third instar larval tissues of *D. melanogaster* *(hsp70-lacZ) Bg* ^*9*^ after exposure to 0.75 µl/ml of MMS for 48 hrs**.**

**Figure 14:**
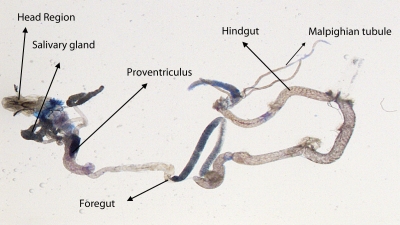
Trypan blue staining pattern in the third instar larval tissues of *D. melanogaster* *(hsp70-lacZ) Bg* ^*9*^ after exposure to 1.0 µl/ml of MMS for 48 hrs.

The results of the present study reveals that the exposure of the third instar larvae of transgenic *Drosophila melanogaster (hsp70-lacZ) Bg* ^*9*^ to different doses of MMS, *i.e.* 0.25, 0.50, 0.75 and 1.0 µl/ml for the duration of 2 hrs did not induce significant expression of *hsp70* as compared to untreated larvae (Table 1; Figure 2). The doses of 0.25 and 0.50 µl/ml MMS showed the effect of exposure duration increase over 4, 24 and 48 hrs on the activity of *hsp70* expression (Table 1; Figure 2). At further higher doses, *i.e.* 0.75 and 1.0 µl/ml, the expression of *hsp70* was significant for different durations of exposure as compared to the untreated larvae but the expression of *hsp70* was less as compared to the treatment of 0.50 µl/ml of MMS for 4, 24 and 48 hrs of exposure (Table 1; Figure 2). Regression analysis was also performed to study the dose effect of third instar larvae of transgenic *Drosophila* *melanogaster (hsp70-lac Z)Bg* ^*9*^ for various durations of exposure (Table 3; Figure 3–6). The exposure to 0.25, 0.50, 0.75 and 1 µl /ml MMS for 4 and 24 hrs was associated with the β-coefficient of 0.327 (F = 0.321) and 0.433 (F = 0.462), respectively (Table 2; Figure 4 and 5). However, for the exposure of 48 hrs, the β-coefficient was – 0.240 (F = 0.124) (Table 2; Figure 6). The reduction in the value of the β-coefficient demonstrates the reduction in β-galactosidase activity for the longest duration of exposure. The regression analysis was also performed to study the effect of exposure durations at various doses of MMS (Table 3; Figure 7–10). The exposure of third instar larvae of transgenic *Drosophila* *melanogaster (hsp70-lac Z) Bg* ^*9*^ to 0.25 µl/ml of MMS for 2, 4, 24 and 48 hrs of duration was associated with the β-coefficient of 0.981 (F = 50.37) (Table 3; Figure 7). Similarly, the exposure of third instar larvae to 0.50 and 0.75 µl/ml MMS for 2, 4, 24 and 48 hrs was associated with the β-coefficient of 0.638 (F = 1.325) and 0.396 (F = 0.371), respectively (Table 3; Figure 8 and 9). The exposure of third instar larvae to 1.0 µl/ml MMS resulted in the reduction of the β-coefficient, *i.e.* 0.261 (F = 0.878) (Table 3; Figure 10). The reduction in the value of the β-coefficient demonstrates the reduction in β-galactosidase activity at the highest dose of exposure. Trypan blue staining was performed to study the tissue damage induced by MMS in the larval tissue exposed to different doses of MMS. About 90% of the untreated larvae were negative to trypan blue staining even after 48hrs of the treatment. In about 80% of the larvae light staining was observed only in the midgut of the larvae exposed to different doses of MMS for 2 hrs but the larvae exposed to higher doses of MMS, *i.e.* 0.75 and 1.0 µl/ml, showed damage in the midgut, salivary glands, malpighian tubules and the hindgut. Figures 11–14) showed trypan blue staining for the control larvae and those exposed to 0.50, 0.75 and 1.0 µl/ml MMS for 48 hrs.

**Table 1:** β-galactosidase activity measured in transgenic *Drosophila melanogaster (hsp70-lacZ) Bg* ^*9*^ third instar larvae exposed to different concentrations of methyl methanesulfonate for various time intervals.

TreatmentsMMS (µl/ml)	After 2 hrsO.D (Mean±SE)	After 4 hrsO.D (Mean±SE)	After 24 hrsO.D (Mean±SE)	After 48 hrsO.D (Mean±SE)
0.25	0.2448±0.0193	0.2425±0.0039	0.2530±0.0520*	0.2740±0.0218*
0.50	0.2106±0.0097	0.3017±0.0114*	0.3100±0.0240*	0.3160±0.0236*
0.75	0.2196±0.0167	0.2865±0.0085*	0.2885±0.164*	0.2740±0.040*
1.0	0.2474±0.0088	0.2718±0.0117*	0.2865±0.0325*	0.2750±0.0382*
Untreated	0.2387±0.0152	0.2355±0.0154	0.2186±0.0125	0.2537±0.0208

* Significant at *p*<0.05 compared to Untreated.

MMS: Methyl methanesulfonate; O.D: Optical density; SE: Standard Error.

**Table 2:** Regression analysis for β-galactosidase activity in the third instar larvae of transgenic *Drosophila melanogaster (hsp70-lacZ) Bg* ^*9*^ to study the dose effect of MMS (0.25, 0.50, 0.75 and 1µl/ml of MMS) for 2, 4, 24 and 48 hrs of exposure.

S.No.	Duration (hrs)	Regression Equation	r-value	-coefficient	SE	*p*-value	F-value
1	2	Y = 0.22640 + 0.00672X	0.11851	0.119	0.027	0.0142	0.28
2	4	Y = 0.25745 + 0.02908X	0.37196	0.372	0.351	0.0181	0.321
3	24	Y = 0.26475 + 0.03160X	0.43324	0.433	0.318	0.0140	0.462
4	48	Y = 0.29540-0.0156X	-0.24160	-0.240	0.030	0.010	0.124

MMS: Methyl methanesulfonate; SE: Standard error

**Table 3:** Regression analysis for β-galactosidase activity in the third instar larvae of transgenic *Drosophila melanogaster (hsp70-lacZ) Bg* ^*9*^ to study the duration exposure effects at fixed concentration.

**S.No.**	**Concentrations (µl/ml)**	**Regression Equation**	**r-value**	**B-coefficient**	**SE**	***p*****-value**	**F-value**
1	0.25	Y = 0.24078 + 0.00066X	0.98072	0.981	0.002	0.0001	50.37
2	0.50	Y = 0.25576 + 0.00148X	0.63836	0.638	0.033	0.0170	1.375
3	0.75	Y = 0.25551 + 0.00060X	0.39586	0.396	0.026	0.0105	0.371
4	1.00	Y = 0.26191 + 0.00042X	0.55252	0.261	0.121	0.0022	0.878

MMS: Methyl methanesulfonate; SE: Standard error

## Discussion

The results of the present study revealed that MMS induced significantly the expression of *hsp70* at 0.25, 0.50, 0.75 and 1.0 µl/ml at 4, 24 and 48 hrs of exposure as compared to the untreated larvae. hsp70 expression was not significant after 2 hrs of exposure. The reduction in the activity of *hsp70* at 0.75 and 1.0 µl/ml of MMS for different times of exposure may be due to a reduction in the number of viable cells after 24 and 48 hrs of exposure or to auto-repression of hsp70 once its upper limit has been achieved. The instability of the reporter gene may also be involved at the exposure to 0.75 and 1.0 µl/ml MMS for different durations that may lead to a decrease in the activity of *hsp70* expression. The tissue damage caused by the exposure to the higher doses of MMS was evident by the trypan blue exclusion assay in the larvae exposed for different durations. A dose-dependent increase in the activity of β galactosidase clearly demonstrated the dose-dependent toxic effect of MMS in transgenic *Drosophila melanogaster (hsp70-lacZ) Bg* ^*9*^ and underlined the usefulness of *hsp70* expression as bio-indicator of exposure to environmental chemicals.

MMS causes DNA damage by methylating N^7^-deoxyguanine and N^3^-deoxyadenine. Methylation causes double-strand DNA breaks and inhibition of replication fork movement. Apart from DNA adduct formation and methylation, MMS also leads to protein adduct formation. MMS methylates the N-terminus of valine and histidine residues in proteins and is thus classified as super clastogen (Zhang *et al.,* 2005). Toxicological studies for MMS have been carried out in various experimental models like mice, rats, etc. According to the National Toxicological Programme guidelines, development and validation of alternative models is necessary to obtain reliable and sensitive results. For traditional toxicological studies a shift has taken place from the use of mammalian models to alternative models and in silico approaches. *Drosophila, Zebra fish, C. elegans* are now used as animal models in toxicological research (Avanesian *et al.*, 2009). *Drosophila* has many similarities with the human genome and is easy to handle, culture, and moreover ethical problems are less serious with this model (AMBR, 2010). Genetically modified models provide reliable information about the mode of action for the test chemical. They provide exactness in toxicological research. The transgenic mouse is already in use for various carcinogenesis studies (Avanesian *et al.,* 2009). *Drosophila melanogaster* has been used in genetic, behavioral and molecular biology research. Recently, *Drosophila* has been used as a model for disease oriented molecular screening. *Drosophila* as a model in pharmaceutical research has been evaluated and validated for various medical problems like aggression, sleep, pain, seizures, psychoactive drug addiction, etc. The use of the alternative *Drosophila* model in pharmaceutical research is time and cost effective in comparison to rodents. In the future *Drosophila* will be used to detect adverse drug reactions. It will also be helpful in reducing time and cost in the field of drug development processes (Avanesian *et al.,* 2009). In the present study, transgenic *Drosophila melanogaster (hsp70-lacZ) Bg* ^*9*^ strain was used to study the effect of MMS on *hsp70* expression and tissue damage in the 3^rd^ instar larvae. Animal models remain important models ranging from worms to primates that can be used for the detection of adverse effects (Avanesia *et al.,* 2009). Although mammalian systems may represent more accurate evaluation tools of short-term and long-term safety, they are frequently laborious and costly, particularly at early stages of drug discovery and development. Application of transgenic models in assaying environmental pollution has opened a new frontier in biomonitoring. Guven *et al.* (1994) and Guven and de Pomerai (1995) have successfully developed transgenic *Caenorhabditis elegans s*train *(hps70-lacZ)* and used it in soil ecotoxicological studies. Halloran *et al.* (2000) cloned zebra fish promoter for the inducible *hsp70* gene and made stable transgenic lines of zebra fish. They express the reporter green fluorescent protein gene under the control of *hsp70* promoter.

The tiny fruit fly or *Drosophila* is a well known model organism for developmental biologists and geneticists. In toxicological arena, however, few reports have successfully employed transgenic *Drosophila* as a model organism in the recent years (Mukhopadhyay *et al.,* 2003). Jowett (1991) showed that the transgenic fruit fly could be used to study both drug metabolism and oxidative stress. The transgenic *Drosophila melanogaster* line that expresses bacterial β-galactosidase as a response to stress was used in the study of Lis *et al.* ( 1983). In the said strain of flies the transformation vector is inserted with a P element; the line contains wild type hsp70 sequence up to the lacZ fusion point. Elevated levels of *hsp70* expression as a measure of cellular assault have been established in the present study. Hence it is concluded that the expression of *hsp70* on exposure to the effect of environmental chemicals is a potential indicator of non-target toxicity. The presented results are suggestive of the cytotoxic potential of methyl methanesulfonate to non target organisms like *Drosophila.* The study further supports the convenient and inexpensive use of *hsp70* expression as a bioindicator of exposure to environmental chemicals.

## References

[CIT0001] AMBR (2010). International symposium on alternate animal models in biological research: present and future perspectives in toxicology.

[CIT0002] Avanesian A, Semnani S, Jafri M (2009). Can Drosophila melanogaster represent a model system for the detection of reproductive adverse drug reactions?. Drug Dis Today.

[CIT0003] Benford DJ, Hanley AB, Bottrill K, Oehlschlager S, Balls M, Brance F, Castegnara JJ, Descotes J, Hemminiky K, Lindsay D, Schilter B (2000). Biomarkers as predictive tools in toxicity testing. The Report and Recommendations of ECVAM workshop 40. Alt Lab Anim.

[CIT0004] Bennett AD, Waters MD (2000). Applying biomarkers research. Environ Health Persp.

[CIT0005] Bierkens JGEA (2000). Applications and pitfalls of stress proteins in biomonitoring. Toxicology.

[CIT0006] Chowdhuri DK, Nazir A, Saxena DK (2001). Effect of three chlorinated pesticides on hsr ( sress gene in transgenic Drosophila melanogaster. J Biochem Mol Toxicol.

[CIT0007] Chowdhuri DK, Saxena DK, Vishwanathan PN (1999). Effect of hexachlorocyclohexane (HCH), its isomers, and metabolites on hsp70 expression in transgenic Drosophila melanogaster. Pesticide Biochem Physiol.

[CIT0008] Cozzi P, Mongelli N, Suarato A (2004). Recent anticancercytotoxic agents. Curr Med Chem Anti-Can Agents.

[CIT0009] Festing MFW, Baumans V, Combes DR, Halder M, Hendricsen FM, Howard BR (1998). Reducing the use of laboratory animals in biomedical research: problems and possible solutions. Alt Lab Anim.

[CIT0010] Fonager J, Beedholm R, Clark BFC, Rattan SIS (2002). Mild stress induced stimulation of heat shock protein synthesis and improved functional ability of human fibroblasts undergoing aging in vitro. Exp Gerontol.

[CIT0011] Gupta SC, Siddique HR, Mathur N, Vishwakarma AL, Mishra RK, Saxena DK, Chowdhuri DK (2007). Induction of hsp70, alterations in oxidative stress markers and apoptosis against dichloruos exposure in transgenic Drosophila melanogaster: modulation by reactive oxygen species. Biochim. Biophys. Acta.

[CIT0012] Guven K, Duce JA, dePomeria DI (1994). Evaluation of a stress inducible transgenic nematode strain for rapid aquatic toxicity testing. Aquatic Toxicol.

[CIT0013] Guven K, de pomerai DI (1995). Differential expression of hsp70 proteins in response to heat and cadmium in Caenorhabditis elegans. J Thermal Biol.

[CIT0014] Halloran MC, Sato-Maeda M, Warren JT, SU F, Lele Z, Krone PH, KU Wada JY, Shoji W (2000). Laser-induced gene expressions in specific cells of transgenic Zebra fish. Development.

[CIT0015] HSDB (2000). http://toxnet.nlm.nih.gov/cgi-bin/sis/search/a?dbs+hsdb:@term+@DOCNO+5103.

[CIT0016] IARC (1987). Overall Evaluations of Carcinogenicity.

[CIT0017] IARC (1974). Some Anti-thyroid and Related Substances, Nitrofurans and Industrial Chemicals.

[CIT0018] Jowett T (1991). Transgenic Drosophila as an in vivo model for studying mammalian drug metabolism. Bioassays.

[CIT0019] Kohler RH, Beltiz B, Eckwert H, Adam R, Rahman B, Trontelj P (1998). Validation of hsp70 stress gene response as a marker of metal effects in Deroceras reticulatum (Pulmonata): Correlation with demographic parameters. Environ Toxicol Chem.

[CIT0020] Krebs RA, Feder ME (1997). Tissue specific variation in hsp70 expression and thermal damage in Drosophila melanogaster larvae. The J Exp Biol.

[CIT0021] Lis JT, Simon JA, Sutton CA (1983). New heat shock puffs and β-galactosidase activity resulting from transformation of Drosophila with an hsp70-lacZ hybrid gene. Cell.

[CIT0022] Lundin C, North M, Erixon K, Walters K, Jenssen D, Goldman ASH, Helleday T (2005). Methyl methanesulfonate (MMS) produces heat-labile DNA damage but no detectable in vivo DNA double-strand breaks. Nucleic Acids Res.

[CIT0023] Merck (1989). The Merck Index, 11th ed.

[CIT0024] Morano KA (2007). New tricks for an old dog: the evolving world of *hsp70*. Ann. New York Academy of Sciences.

[CIT0025] Mukhopadhyay I, Nazir A, Mahmood K, Saxena DK, Das M, Khanna SK, Chowdhuri DK (2002). Toxicity of argemone oil: Effect on hsp70 expression and tissue damage in transgenic Drosophila melanogaster (hsp70 lac Z) Bg^9^. Cell Biol Toxicol.

[CIT0026] Mukhopadhyay I, Saxena DK, Chowdhuri DK (2003). Hazardous effects of effluent from the chrome plating industry: 70kDa heat shock protein expression as a marker of cellular damage in transgenic Drosophila melanogaster (hsp70 lac Z). Environmental Health Perspective.

[CIT0027] Mukhopadhyay I, Nazir A, Saxena DK, Chowdhuri DK (2003). Heat shock responses: hsp70 in environmental monitoring. J Biochem Mol Toxicol.

[CIT0028] Nazir A, Mukhopadhyay I, Saxena DK, Siddiqui MS, Chowdhuri DK (2003). Evaluation of toxic potential of captan: Induction of hsp70 and tissue damage in transgenic Drosophila melanogaster (hsp70-lacZ) Bg^9^. J Biochem Mol Toxicol.

[CIT0029] Nazir A, Mukhopadhyay I, Saxena DK, Chowdhuri DK (2001). Chorpyrifos induced hsp70 expression and effect on reproductive performance in transgenic Drosophila melanogaster (hsp70-lacZ)Bg^9^. Arch. Environ. Contam. Toxicol.

[CIT0030] Nover L (1984). Heat shock response of eukaryotic cells.

[CIT0031] Nover L (1991). The heat shock response.

[CIT0032] Ritossa F (1996). Discovery of the heat shock response. Cell Stress Chap.

[CIT0033] Siddique YH, Ara G, Afzal M (2011a). Effect of ethinylestradiol on hsp70 expression in transgenic. Drosophila melanogaster (hsp70-lacZ) Bg^9^ Pharmacologyonline.

[CIT0034] Siddique YH, Ara G, Afzal M (2011b). Effect of cyclophosphamide on hsp70 expression in transgenic Drosophila melanogaster (hsp70-lacZ) Bg^9^ Drosophila Inf Ser.

[CIT0035] Siddique HR, Gupta SC, Dhawan A, Murthy RC, Saxena DK, Chowdhuri DK (2005). Genotoxicity of industrial solid waste leachates in Drosophila melanogaster. Environ. Mol. Mutagen.

[CIT0036] Tavaria M, Gabriele T, Kola I, Anderson RL (1996). A hitchhiker's guide to the human hsp70 family. Cell Stress Chap.

[CIT0037] WHO (1993). Guidelines for Drinking Water Quality, 2nd Ed.

[CIT0038] Yoda K, Shimizu M, Fujimura S (1982). Induction of morphological differentiation in cultured mouse neuroblastoma cells by alkylating agents. Carcinogenesis.

[CIT0039] Zhang F, Bartels MJ, Pottenger LH, Bhaskar B, Gollapudi BB (2005). Differential adduction of proteins vs. deoxynucleosides by methyl methanesulfonate and 1-methyl-1-nitrosourea in vitro. Rapid Comm Mass Spec.

